# Computation and visualization of cell–cell signaling topologies in single-cell systems data using *Connectome*

**DOI:** 10.1038/s41598-022-07959-x

**Published:** 2022-03-09

**Authors:** Micha Sam Brickman Raredon, Junchen Yang, James Garritano, Meng Wang, Dan Kushnir, Jonas Christian Schupp, Taylor S. Adams, Allison M. Greaney, Katherine L. Leiby, Naftali Kaminski, Yuval Kluger, Andre Levchenko, Laura E. Niklason

**Affiliations:** 1 Department of Biomedical Engineering, Yale University, New Haven, CT USA; 2 Medical Scientist Training Program, Yale School of Medicine, New Haven, CT USA; 3 Applied Mathematics Program, Yale University, New Haven, CT USA; 4 Section of Pulmonary, Critical Care, and Sleep Medicine, Yale School of Medicine, New Haven, CT USA; 5 Department of Pathology, Yale University, New Haven, CT USA; 6 Interdepartmental Program in Computational Biology and Bioinformatics, Yale University, New Haven, CT USA; 7 Yale Systems Biology Institute, Yale University, West Haven, CT USA; 8 Department of Anesthesiology, Yale School of Medicine, New Haven, CT USA; 9 NOKIA Bell-Laboratories, Murray Hill, NJ USA

**Keywords:** Stem cells, Computational biology and bioinformatics, Data mining, Data processing, Gene regulatory networks, Network topology, Software, Statistical methods, Systems biology, Systems analysis

## Abstract

Single-cell RNA-sequencing data has revolutionized our ability to understand of the patterns of cell–cell and ligand–receptor connectivity that influence the function of tissues and organs. However, the quantification and visualization of these patterns in a way that informs tissue biology are major computational and epistemological challenges. Here, we present *Connectome*, a software package for R which facilitates rapid calculation and interactive exploration of cell–cell signaling network topologies contained in single-cell RNA-sequencing data. *Connectome* can be used with any reference set of known ligand–receptor mechanisms. It has built-in functionality to facilitate differential and comparative connectomics, in which signaling networks are compared between tissue systems. *Connectome* focuses on computational and graphical tools designed to analyze and explore cell–cell connectivity patterns across disparate single-cell datasets and reveal biologic insight. We present approaches to quantify focused network topologies and discuss some of the biologic theory leading to their design.

## Introduction

Cell-to-cell communication is a major driver of cell differentiation and physiological function governing organ development, homeostasis, and response to injury. Within tissues, cells have local neighbors with whom they directly communicate via paracrine signaling and direct cell–cell contact, and long-range or mobile partners with whom they exchange information via endocrine signaling. In solid tissues, cell types have specific cellular niches incorporating localized matrix and signaling environments, which facilitate phenotypic maintenance and support specialized cell functions. Circulating immune cells use an extensive library of chemokines to coordinate multicellular system responses to threat or injury. The advent of single-cell technologies has made it technologically possible, for the first time, to codify cell-specific ligand–receptor patterns in complex tissues with both high accuracy and robust statistical confidence. Numerous computational tools have emerged in the last years to mine intercellular communication information from single-cell data^1–8^. The combination of single-cell sequencing data with ligand–receptor mapping is therefore a promising approach to exploring, understanding, and reverse-engineering complex tissue systems-biology for biologic, therapeutic, and regenerative efforts. This manuscript formalizes and disseminates the techniques first published by Raredon et al.^1^, presenting open-source software to the wider community facilitating exact recapitulation of this analysis.

A connectomic network from a single tissue has unique properties that must be taken into consideration for biologically relevant downstream information processing. Tissue-derived connectomic networks are directional—i.e. each ligand–receptor interaction matrix is asymmetric; multi-modal—i.e. many ligand–receptor mechanisms contribute to the connectome; and weighted—i.e. interaction edges can be assigned quantitative values. These properties make data mining and data visualization substantially more complex than in some other genres of network science. Added dimensions can additionally come into play when it is necessary to compare cell–cell signaling in tissues between experimental conditions, over time during growth or remodeling, or between disparate tissue systems in which the same cell type annotations are not necessarily present. Here we describe a computational package in R called *Connectome* which facilitates each of these tasks*. Connectome* has been used to explore native signaling in human lung^9^ and to identify aberrant signaling in pulmonary arterial hypertension (PAH)^10^, chronic obstructive pulmonary disease (COPD)^11^, and COVID-19^12^.

*Connectome* is a multi-purpose tool designed to create ligand–receptor mappings in single-cell data, to identify non-random patterns representing signal, and to provide biologically-informative visualizations of these patterns. It can be applied to any single-cell dataset (or combination of datasets), and is designed to be used in association with the R package *Seurat*. By default, *Connectome* uses the FANTOM5 database of ligand–receptor interactions^13^, formalizing the workflow first presented in^1^, but it also allows mapping against any user-provided ligand–receptor list. Because the reference database can be customized, *Connectome* can also be used to investigate newly discovered or hypothesized ligand–receptor mechanisms of particular interest.

To demonstrate *Connectome*, we apply the software analysis in three distinct use-cases on public data. First, we demonstrate application to an individual tissue by analyzing single-cell human pancreas data. Second, we describe differential connectomics, comparing IFN-stimulated human PBMCs with unstimulated control data. Third, we apply *Connectome* to a longitudinal wound-healing dataset in mouse muscle. All software is available at https://github.com/msraredon/Connectome. Detailed vignettes and instructions for use are published at https://msraredon.github.io/Connectome/.

## Methods

### Definitions

The term *connectome* refers to the complete set of interactions between nodes in a cell system, in the same way that *transcriptome* refers to the complete set of transcribed genes within a cell. The term *parcellation* refers to the way that the above system is divided up into distinct nodes and in the application described in this manuscript is synonymous with *celltype cluster*. The way a system is parcellated strongly affects its node architecture and therefore the shape of the resulting connectome. An *edge* in *Connectome* is a single unique celltype–ligand–receptor–celltype interaction. *Edge attributes* are quantitative or qualitative pieces of information associated with an edge. A *differential connectome* is the network that results when two connectomes are directly compared, edge-for-edge. *Centrality*, as used in the text, refers to quantitative metrics of how ‘connected’ a given node is to other nodes, in either an outgoing (sending) or incoming (receiving) fashion.

### Defining the data structure of tissue connectomics

The connectomic mapping discussed here treats every cell parcellation (e.g., different cell types, phenotypically distinct cell states of the same cell type, etc.) as a single node, averaging ligand and receptor values across a given cell parcellation to yield mean values which are then linked. Mean-wise connectomics has the advantage of accommodating the zero-values intrinsic to single-cell data, while simplifying the system so that every cell parcellation is represented by a single, canonical node. However, as this approach will blend the effects of all cellular archetypes within a cluster, initial cell parcellation must be done carefully for the resulting connectomic networks to be biologically meaningful.

*Connectome*, by default, calculates two distinct edgeweights, each of which captures biologically relevant information. The first edgeweight, also referred to as ‘w_1_’ or ‘weight_norm_,’ is defined as the product of the celltype-wise normalized expression for the ligand and the receptor, or

$$ w_{1}^{ijk} = weight_{norm}^{ijk} = product \left(Celltype_{i}^{{L_{k}^{normalized} }} ,Celltype_{j}^{{R_{k}^{normalized} }} \right) $$

where (k) denotes a specific ligand–receptor pair, $Celltype_{i}^{{L_{k}^{normalized} }}$ is the normalized expression of ligand $L_{k}$ in celltype (i), and $Celltype_{j}^{{R_{k}^{normalized} }}$ is the normalized expression of receptor $R_{k}$ in celltype (j). Weight_norm_ is useful for differential connectomics, in which exact edges are compared across tissue conditions. However, Weight_norm_ is encumbered by the fact that it ignores the cell-type *specificity* of many ligands and receptors. Weight_norm_ will weight a highly expressed but non-specific cell–cell link as stronger than a highly specific but lowly-expressed gene. This does not align with biologic intuition for many cell–cell signaling mechanisms, nor with the goal of parsing out rare cell types that may have outsized biological relevance. Therefore, *Connectome* also calculates a second edgeweight, alternatively referred to as ‘w_2_’ or ‘weight_scale_,’ which is defined as the mean of the gene-wise z-scores of the ligand and receptor, or

$$ w_{2}^{ijk} = weight_{scale}^{ijk} = mean \left(Celltype_{i}^{{L_{k}^{scaled} }} ,\; Celltype_{j}^{{R_{k}^{scaled} }} \right) $$

where $Celltype_{i}^{{L_{k}^{scaled} }}$ is the system-scaled expression of ligand $L_{k}$ in a celltype (i) and $Celltype_{j}^{{R_{k}^{scaled} }}$ is the system-scaled expression of receptor $R_{k}$ in a celltype (j). Because weight_scale_ incorporates information on the topology of the entire system being analyzed, leveraging system z-scores of each gene, it is ideally suited to exploring cellular ‘roles’ within a single tissue system. It also can be used to compare cellular prominence within signaling networks across disparate tissue systems, species, or batches, in which normalized values may vary but their *relative* expressions across cell types do not^1^.

Alternative edgeweights, such as those incorporating a score for likelihood of downstream signal transduction^5^ or those which take into account proximity information from spatial transcriptomics or histology-registered techniques^14^, may reveal additional patterns. For the purposes of this software program, *Connectome* defaults to the above edgeweights definitions because we have found them to be effective for understanding biological questions addressed in our studies.

### Identification of edges of interest or significance

Statistical significance depends upon the question being asked, the test being applied, and the threshold for important differences, and has a very different meaning depending on whether a research team is investigating a single tissue, comparing multiple tissues, or studying a physiologic process. We find two general statistical patterns to be of key importance, and we have built-in functionality to *Connectome* to help the researcher focus on these patterns.

#### Single-tissue system

First, when studying a single tissue system (i.e., one organ in one condition), it is desirable to focus on those ligand–receptor interactions which are highly associated specific celltype-celltype pairings. To do so, we recommend limiting analysis to only those edges where the ligand and the receptor are both expressed in greater than a certain fraction of their respective clusters, generally 10%, and to then further limit analysis to those edges where both the ligand and the receptor have a p-value of less than a given threshold as determined by a system-wide Wilcoxon Rank Sum Test (calculated by default within *CreateConnectome*.) These thresholds can be further reduced within *FilterConnectome,* to identify those pathways of greatest significance, and can be crossed with additional thresholding to limit edge representation to specific vectors or signaling families (see vignettes online).

#### Two-tissue comparison

When comparing two tissue systems (i.e., one organ in two experimental conditions) it is generally interesting to focus on those mechanisms which, regardless of their association with a specific celltype-celltype vector, are differentially regulated across condition.

The vignette online shows how to use a Wilcoxon Rank Sum test to compare each pair of commensurate cells across two datasets and to thereby determine which ligands and which receptors are differentially expressed to a statistically significant degree. The differential connectome can then be thresholded to only those edges where both the ligand on the sending cell and the receptor on the receiving cell are differentially expressed between experimental conditions. All differential connectomics presented and discussed in this manuscript and online utilize this statistical approach.

### Centrality definition and usage

*Centrality* and *CompareCentrality* both calculate and plot-to-compare two key centrality metrics for networks of interest: the cumulative incoming/outgoing edgeweight for each node, and the Kleinberg Hub and Authority scores for each node. These two centrality metrics often correlate, but they do not always agree on node ranking. There is no single, proper and agreed upon way to best calculate network centrality^16^; these two metrics were chosen because they are commonly used in network research and are biologically interpretable. Cumulative incoming/outgoing edgeweight fraction is the sum of the incoming and outgoing edges, calculated per node, expressed by default as the fraction of total edgeweight within the considered system; this ranks each node’s ability to contribute or listen to a given set of interactions. Hub-Authority centrality is a heavily-used information flow metric^17^, which should be interpreted, in this instance, to reveal Hubs which send signal to Authorities, and Authorities which receive information from Hubs. Each of these metrics provide interpretable information regarding biologic network structure.

### Perturbation score definition and usage

In a differential connectome, every edge may fall into one of four distinct categories, ligand UP/receptor UP, ligand DOWN/receptor DOWN, ligand UP/receptor DOWN, or ligand DOWN/receptor UP. If both the ligand and receptor are upregulated, we may reasonably call the edge ‘activated’, while conversely, when both the ligand and receptor are downregulated, we may think of the edge as ‘deactivated’. The two alternate cases, when a ligand is upregulated and a receptor down, or vice-versa, are more complicated to interpret. Although such patterns may initially suggest ligand–pressure or ligand–starvation, in which the downstream cell changes its character in response to upstream influence, it is important to note that in most tissue systems there are many cells interacting at once, and that there are likely multi-cell feedback loops in play which confound easy interpretation of such patterns.

To accommodate this data architecture and identify strongly perturbed edges regardless of category, we defined a ‘perturbation score’ that is calculatable for each edge, expressed as the product of the absolute values of the log-fold change for both the receptor and ligand, or:

$$ score^{ijk} = \left| {log_{2} \left( {\frac{{Celltype_{i}^{{L_{k}^{test} }} }}{{Celltype_{i}^{{L_{k}^{control} }} }}} \right)} \right| \times \left| {log_{2} \left( {\frac{{Celltype_{j}^{{R_{k}^{test} }} }}{{Celltype_{j}^{{R_{k}^{control} }} }}} \right)} \right| $$

where $Celltype_{i}^{{L_{k}^{test} }}$ and $Celltype_{i}^{{L_{k}^{control} }}$ are the normalized expression of the ligand in celltype (i) in the test and control condition, respectively, and $Celltype_{j}^{{R_{k}^{test} }}$ and $Celltype_{j}^{{R_{k}^{control} }}$ are the normalized expression of the receptor in celltype (j) in the test and control condition, respectively. The perturbation score is calculated in the above fashion so that edges with large changes on both the ligand and receptor side, regardless of sign, have very high scores, and minimally perturbed edges have a score of zero. The perturbation score therefore varies from 0 to Infinity, and increases proportionally to both the log fold-change of the ligand (in either direction) and to the log fold-change of the receptor (in either direction). It is generally useful to limit analysis to those edges which have ligand and receptor expression in > 10% of their respective clusters in either the control or the test condition to avoid noisy measurements; this thresholding functionality is built into *CircosDiff* and *DifferentialScoringPlot*.

### Signaling family categorization

The signaling family categorizations used in this manuscript are carried over from^1^ with minor updates per recent literature findings. These groupings are loaded by default within *Connectome.* It should be noted that each signaling mechanism is allowed only a single designation in this formulation; in biologic reality, however, many signaling mechanisms can be considered to belong to multiple signaling families. These groupings are meant as a guide for hypothesis generation and later downstream exploration, rather than as definitive classification.

### Analysis of pancreas data

In brief, the panc8 data was downloaded from *SeuratData*, normalized, scaled, and run through the *CreateConnectome* with a min.cells.per.ident cutoff of 75. Downstream analysis was limited to those edges where both the ligand and the receptor were expressed in > 10% of their respective clusters, and which had a p-value < 0.05 for both the ligand and the receptor. Additional thresholds applied for specific visualization purposes are emphasized when appropriate in the figure legends.

For the centrality analysis present in Fig. 2, *Centrality* was first run across all signaling families. Each signaling family was then grouped according to which of the 4 cell classes was found to dominate incoming centrality within the given dataset. *Centrality* was then re-run 4 times, once for each set of signaling families, to yield the 4 sub-graphs.

### Analysis of IFN-stimulated vs. Control PBMC data

In brief, the ‘ifnb’ dataset was loaded from *SeuratData.* Each dataset was normalized, scaled, and run through *CreateConnectome*. The two connectomes were then passed to *DifferentialConnectome.* A Wilcoxon Rank Sum test was then performed for each individual cell across conditions, for all ligands and all receptors, and the results from this test were used to limit the differential connectome to only those edges showing statistically significant differences (p < 0.05) for both the ligand and the receptor. Downstream analysis was further limited to those edges which had ligand and receptor expression in > 10% of their respective clusters in *either* the control or the test condition. Figure 3C was made with *DifferentialScoringPlot* and Fig. 3D was made with *CircosDiff*, both of which are built in to *Connectome.*

### Analysis of muscle wound-healing data

In brief, raw data was loaded from De Micheli et al.^18^, normalized, scaled, and run through *CreateConnectome*. *ggplot* was use for Fig. 4A,D, *CircosPlot* for Fig. 4B, and *CompareCentrality* for Fig. 4C.

## Results

### Visualizing the connectomic signature of a single tissue

**Figure 1:**
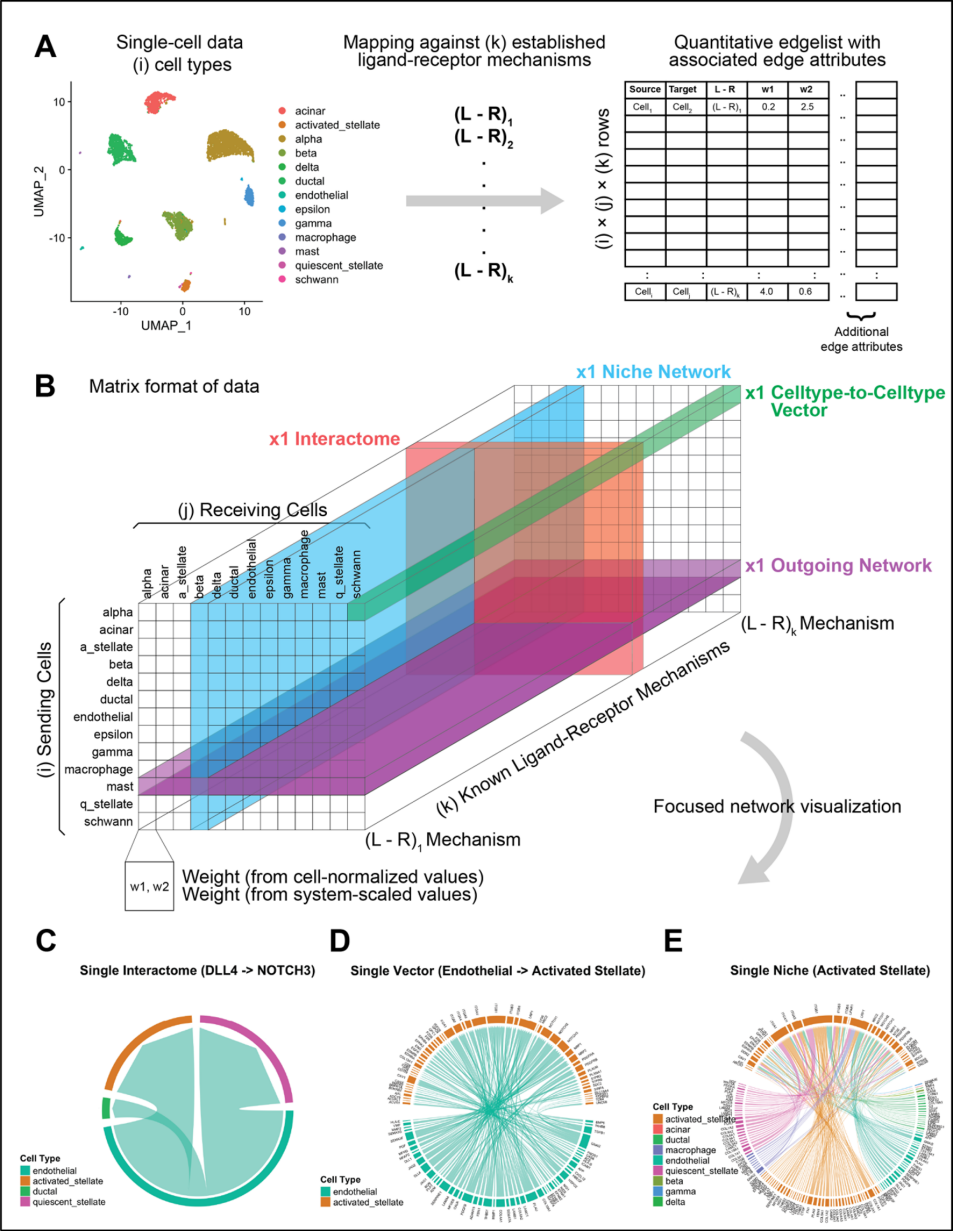
Single-tissue connectomic topology. (**A**) Pancreas single-cell data is parcellated into defined cell types. These cell types are then mapped against a ground-truth database of known ligand–receptor mechanisms. This yields a comprehensive edgelist of cells expressing ligand connecting to cells expressing receptor, with associated edge attributes. Note that a single ligand may hit multiple receptors and vice-versa. (**B**) A conceptual visualization of the data architecture and biologically informative cuts through the data. (**C**–**E**) Selected quantitative visualizations of interactome-, vector-, and niche-networks made with *Connectome.* These three plots, respectively, allow (**C**) identification of top cell types utilizing a given L–R mechanism, (**D**) top mechanisms employed by a cell–cell vector, and (**E**) top cell-mechanism combinations in a position to influence a receiving cell. In (**C**)–(**E**), edge thickness is proportional to weight_scale_, which is larger when an edge is more highly associated with a specific celltype–celltype vector. In all cases, the network shown has been limited to those edges in which the ligand and receptor are both expressed in > 10% of their respective clusters and have a p-value of < 0.05. In subpanel E, for illustration, the network has been further thresholded to those edges with a ligand and receptor z-score of > 1. Connectome v1.0.0 (available at github.com/msraredon/Connectome) was used to generate subfigures (**C**)–(**E**).

Figure 1 shows the workflow for ligand–receptor connectomics in a single tissue. In this instance we use single-cell data from 8 separate single-cell RNA libraries of human pancreatic tissue, available through the *SeuratData* database^19^. As a first step (Fig. 1A), the tissue data are parcellated into defined cell types using a standard clustering workflow. Then, a mapping is created against a ground-truth database of known ligand–receptor mechanisms. This mapping yields a large edgelist, wherein nodes are defined as cell types and edge attributes contain quantitative information derived from cell type-specific expression levels. Source nodes denote the ligands, and target nodes refer to the cognate receptors. This data architecture is stored as a data frame in R, the environment which is particularly amenable to subsetting networks of interest and working with graph theory-based computational packages. This process is performed by a single function in *Connectome* which allows customization of edgeweight definition and edge attribute calculations. By default, two edgeweights are calculated: weight_norm_ and weight_scale_, both of which are defined in “Methods”. Weight_norm_ is a measure of raw connectivity between two nodes, while weight_scale_ is a measure of signal specificity between two nodes within a given tissue system. Both metrics are biologically informative.

Conceptually, the data architecture for a single edge attribute (one column in the above discussed edgelist data frame in Fig. 1A) can be thought of as a 3D matrix (Fig. 1B), where rows are source (sending) cells, columns are target (receiving) cells, and the z-axis is the full list of ground-truth known ligand–receptor mechanisms. This allows clear visualization of the data that needs to be subset in order to explore a single interactome (red), outgoing network (purple), niche network (blue), or cell–cell vector (green). The blue rectangle represents a niche-network, containing information on all edges in a position to influence a single cell type. The purple rectangle shows all edges coming from a single cell type. The red rectangle is a single interactome, containing information for a single signaling modality between all cell types. The green prism represents a single cell-to-cell vector, containing information for all signaling modalities.

Visualization of these connectomic networks can be done in multiple ways. *Connectome* includes a series of functions designed for tissue network exploration, including one which generates plots allowing immediate quantitative visualization of individual interactomes (e.g. Fig. 1C), celltype-to-celltype vectors (Fig. 1D) and niche networks (Fig. 1E). Further, the similarity between individual celltype-to-celltype vectors (vectortypes) can be analyzed using a k-nearest-neighbor style embedding (Fig. S1). This style of visualization places vectortypes in a 2-dimensional space and provides a quantitative way to cluster vectortypes based on which ligand–receptor mechanisms are most highly weighted in each celltype-to-celltype pairing. We provide a custom function in *Connectome* to perform this analysis.

### Tissue network centrality analysis

**Figure 2:**
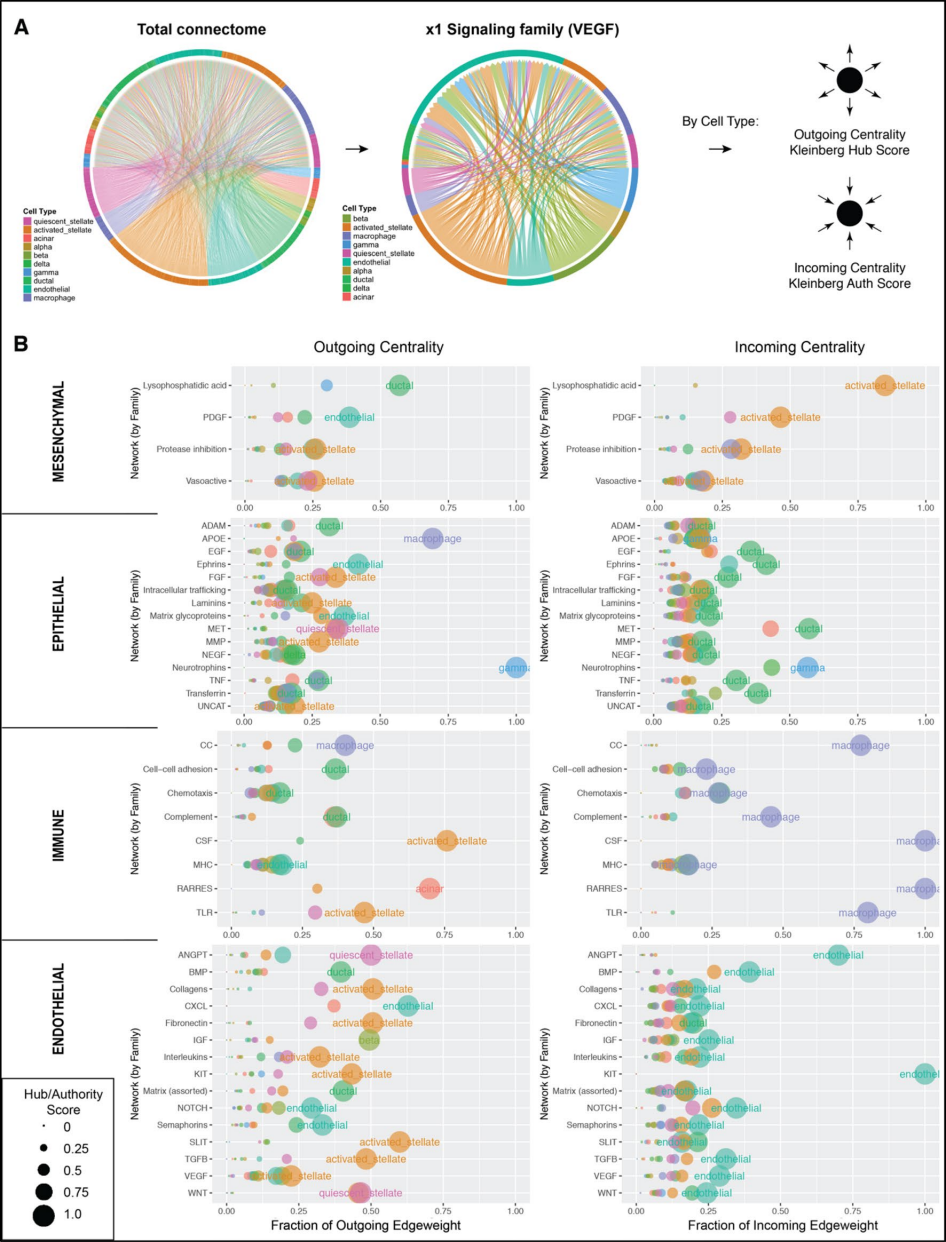
Centrality analysis for a single tissue system. (**A**) The total connectome for the pancreas is iteratively subset to each individual signaling family, after which Kleinberg hub and authority scores are calculated for each cell type. (**B**) Signaling families, grouped by dominant receiving nodes, crafts a quantitative portrait of global tissue system signaling architecture. In alignment with biologic intuition, mesenchymal cells are top network receivers of PDGF-family signals, epithelial cells are top network receivers of EGF-family signals, immune cells are top networks receivers of CSF- and CC (Chemokine)-family signals, and endothelial cells are in top network receiving positions for VEGF-, and ANGPT-family pathways. Centrality analysis was performed once for all signaling families, groupings were designated by top receiving node, and the four grouped plots were then individually re-made. Connectome v1.0.0 (available at github.com/msraredon/Connectome) was used to generate subfigures (**A**) and (**B**).

A major goal of tissue science and cell biology is to understand the roles that individual cells types play and their ability to affect other cell types. It is of interest, therefore, to rank cell types based on their ability to produce or receive information within a given signaling network, or signaling family. To quantify these roles, *Connectome* is capable of performing a centrality analysis, an example which is shown in Fig. 2. In this analysis, the total connectome for a single tissue is first subset down to only those edges belonging to a single signaling family (Fig. 2A). This weighted graph is then used to calculate two centrality metrics: the Kleinberg Hub and Kleinberg Authority scores (represented as the dot size), and the cumulative directed edgeweight (x-axis), for each cell type within the network. A high Hub score, biologically, implies that a sending cell type is producing high levels of ligand which are sensed by other cells in the system. A high Authority score, biologically, means that a receiving cell type is highly expressing receptors capable of sensing ligands secreted by other cells in the system. The dot sizes arising from the Kleinberg scores are then used to visualize outgoing and incoming centrality of each cell type (Fig. 2B), for a variety of specific signaling mechanisms. For example, the endothelial cell population of the pancreas has a high outgoing centrality score for signaling along the PDGB axis to the mesenchyme compartment (green circle in upper left panel of Fig. 2B, “endothelial”, highly connected to the ochre circle in upper right panel, ‘activated stellate’). The result is a ranking of outgoing and incoming centrality for every cell type, across every signaling family, for each target cell type within the tissue. Collectively, this analysis creates a comprehensive portrait of potential extracellular signal transfer within a given tissue system. As shown in Fig. 2, this information can then be used to group each signaling family based on the cell type that is best positioned to receive information (shown, sorted by incoming centrality) or best positioned to generate a signal (not shown—requires sorting by outgoing centrality).

### Differential connectomics with aligned nodes

**Figure 3:**
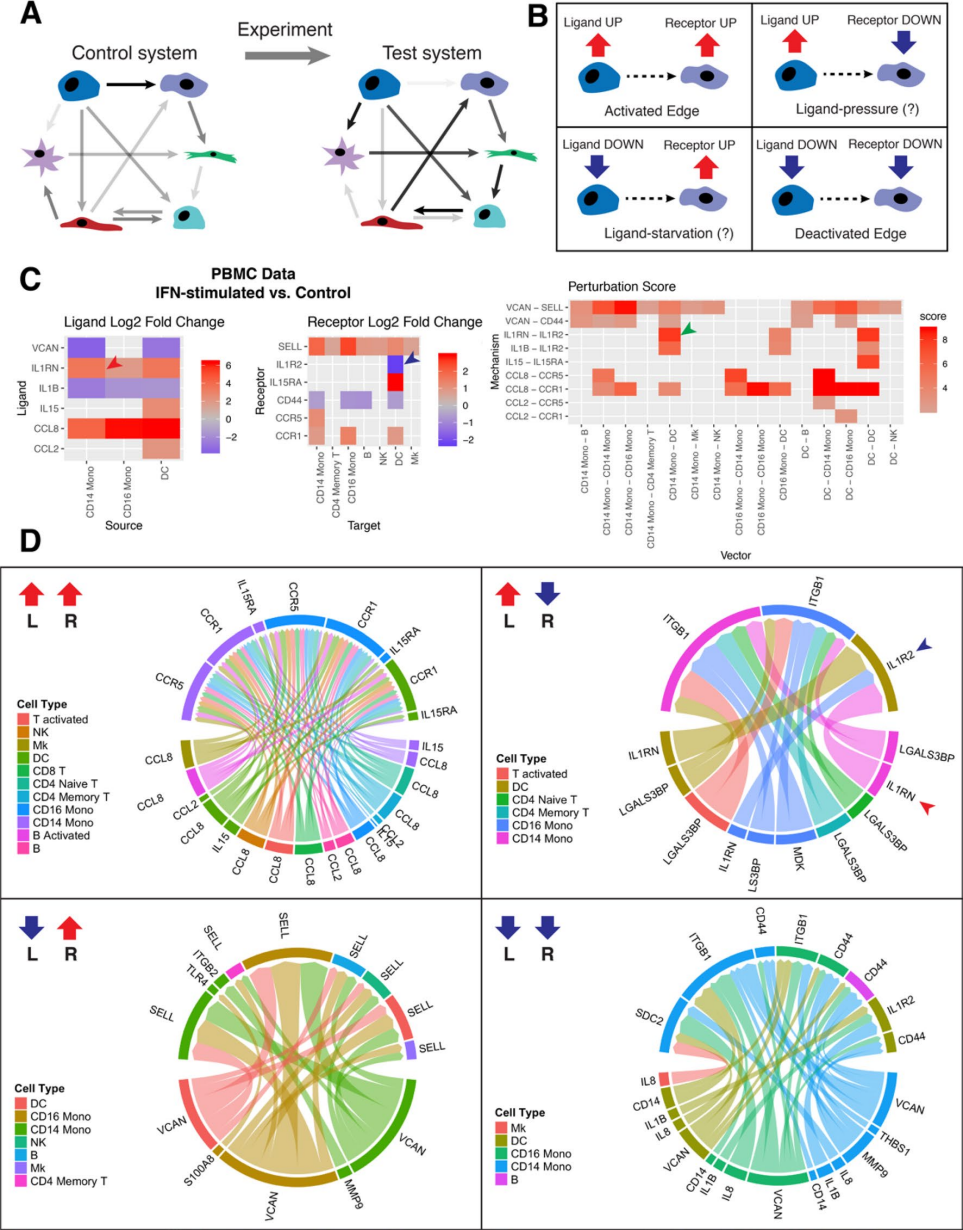
Differential tissue connectomics. (**A**) Schematic showing comparison of a perturbed multicellular system against a known control or reference set of interactions. (**B**) Assuming we are only interested in those edges in which either the ligand or the receptor changes due to perturbation, each edge in a differential systems comparison falls into one of four distinct styles: the ligand and receptor are either both up, both down, or some combination. (If edges are also to be considered in which *only* the ligand or receptor change, then there are eight distinct categories of edge shift.) Dual ligand/receptor increase or decrease are consistent with edge activation or deactivation, respectively. A decreased ligand paired with an elevated receptor suggests ligand starvation, as is often seen in in vitro experiments. And increased ligand paired with a decreased receptor suggests the converse, ligand pressure. (**C**) Application to IFN-stimulated versus control PBMC data, showing how a positive ligand fold change (red arrow) and negative receptor fold change (blue arrow) combine to form a single positive edge perturbation score (green arrow). For illustration purposes, the differential network here has been heavily thresholded (minimum perturbation score of 2, a minimum expression cutoff of 20%, and only significant edges) yielding the presence of grey squares and the low number of displayed nodes and mechanisms. (**D**) Shows differential cell–cell signaling in IFN-stimulated PBMCs versus controls, sorted by style of perturbation. Edge thickness is proportional to perturbation score. Edge color correlates with source celltype. Blue and red arrow-heads emphasize the same edge similarly emphasized in (**C**). Networks in (**D**), for illustration, have been thresholded to a minimum perturbation score of 2 and a minimum percentage cutoff of 10%. In all cases, differential network analysis has been limited to those edges where the expression of both the ligand and receptor, in their respective populations across condition, are differentially expressed with a p-value < 0.05 as assessed by a Wilcoxon Rank Sum test. Connectome v1.0.0 (available at github.com/msraredon/Connectome) was used to generate subfigures (**C**) and (**D**).

It is common in systems biology to examine changes in cell–cell signaling between two systems, i.e., in a multicellular tissue before and after treatment with a drug or chemokine (Fig. 3A). In one such example, if the same cell types are present in both systems, we may calculate direct, one-to-one comparison of all edges using the *Connectome* package. It should be noted, however, that within a differential connectome, there are *four* distinct types of perturbed edges, since for each cell in a differential edge, the ligand and the receptor may be either increased or decreased (see “Methods”, Fig. 3B). Figure 3 shows the utility of this concept in the interferon-simulated vs. control dataset of human peripheral blood mononuclear cells available through *SeuratData*^20^. Figure 3C shows log fold changes for selected ligands and receptors, and the resulting perturbation scores (which are always positive) for each cell–ligand–receptor–cell edge. Perturbed edges can then be grouped by their differential pattern and their perturbation scores visualized in readily-explorable network form (Fig. 3D).

### Longitudinal connectomics for a dynamic tissue-system process

Comparing tissue-level connectomics datasets can be difficult when the cell nodes are not directly comparable between the two tissue systems. Such a situation can occur if new cell types are recruited into, or eliminated from, a tissue system. Alternatively, normal differentiation processes may take place which cause a new cell type to emerge. Such changes occur frequently, whether due to inflammation, response to injury, wound healing, or normal development. Many unanswered questions in tissue science center around how these changing cellular landscapes correlate with shifting cell–cell communication patterns that are present in tissues and organs. Because centrality analysis quantifies network topology while being agnostic to specific nodes being present, the described technique allows for the comparison of disparate tissue systems which do not necessarily contain the same cell types.

**Figure 4:**
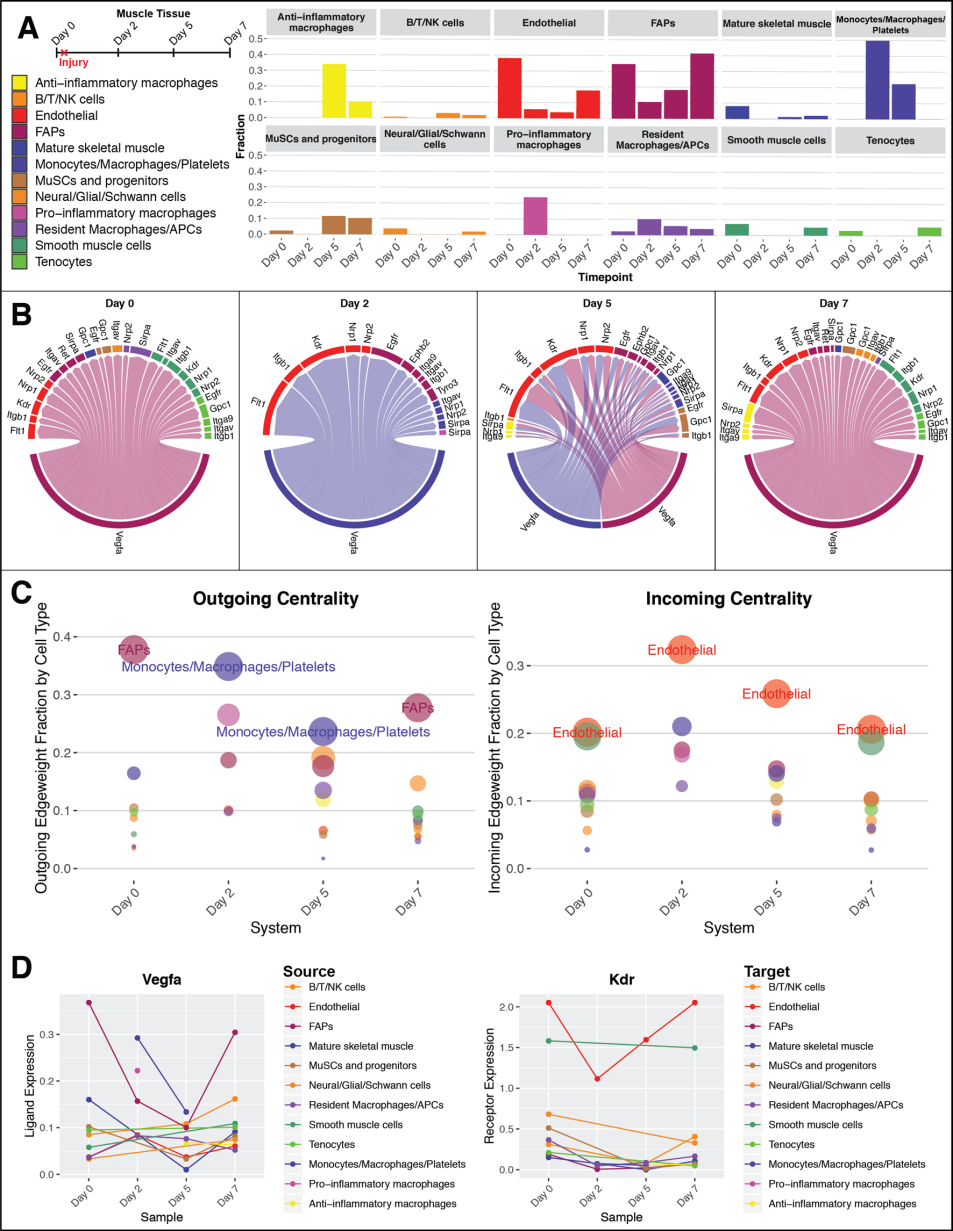
VEGFA signaling over time in healing muscle tissue. (**A**) Schematic of in vivo muscle injury experiment from De Michelis et al.^18^ and associated dynamics in cell type dissociation fraction. (**B**) Vegfa signaling networks within muscle tissue at each time point. Edges coming from fibro/adipogenic progenitors (FAPs) are colored maroon while edges coming from monocytes/macrophages are colored blue. These network plots have been thresholded (minimum 10% expression) for legibility: the only source nodes which meet this criterion are the FAPs in Day 0, 5, and 7 and the Monocyte/Macrophage cluster in Days 2 and 5. Edge thickness is proportional to weight_scale_. (**C**) Centrality analysis, over time, for all Vegfa-mediated edges between all cell types, without any thresholding. Recruited monocytes take over dominant Vegfa production in healing tissue from homeostatic fibro/adipogenic progenitors. Endothelial cells are consistently in the dominant position to receive information, through their expression of multiple receptors including Kdr, Flt1, and Nrp1/2. (**D**) Normalized expression of Vegfa (ligand) and Kdr (receptor) for all cell types over time, showing relative expression. This analysis can be generalized to any network made from any combination of ligand–receptor mechanisms. For simplicity we show an example here based on only a single ligand. Connectome v1.0.0 (available at github.com/msraredon/Connectome) was used to generate subfigures (**B**)–(**D**).

As an example of a comparison of tissue systems containing different cell types, the muscle wound-healing dataset recently published by De Micheli et al.^18^ provides an excellent use-case for longitudinal tissue connectomics. In this study, done in vivo in mice, muscle tissue was injured and then allowed to heal over time. scRNAseq was performed on Day 0, immediately before injury, and then on Days 2, 5, and 7 post-injury. Cells were parcellated on a per-time-point basis, leading to clear trends in cell type tissue fractions over time (Fig. 4A). Certain cell types are present in the tissue throughout the wound healing process [i.e., endothelial cells, fibro/adipogenic progenitors (FAPs)], while others are recruited into the tissue solely during acute wound healing (i.e., monocytes/macrophages/platelets).

For demonstration, we explore the network topology of cell–cell signaling based on Vascular Endothelial Growth Factor A (Vegfa) over time. Figure 4B–D suggests that there is a dramatic change in the dominant source of this ligand during wound healing. In the baseline state, FAPS are the dominant producers of Vegfa. Immediately after injury, FAPs reduce their production of this ligand, and newly recruited Monocytes/Macrophages/Platelets take up dominant production of Vegfa. On Day 5, these two cell types share this functional role, and at the conclusion of healing, FAPs again dominate the network. Endothelial cells, meanwhile, are always expressing a panoply of receptors for this secreted factor and are in a prime position to receive angiogenic information. We see that supporting cell types, including smooth muscle cells and tenocytes, are also in a position to receive Vegfa-mediated information before injury and after the conclusion of healing (green sectors). Further, we observe that muscle stem cells (MuSCs) are also capable of sensing aspects of Vegfa-mediated signaling, in particular on Day 5 post-injury, when they co-express and upregulate established angiogenesis-modulating receptors Egfr, Gpc1, and Itgb1^21–23^. It should be noted that, because of the parcellations chosen, this technique cannot necessarily tell the difference between an entire population of cells shifting in character versus a new phenotypic archetype emerging within an existing population. Further sub-clustering (i.e. finer, follow-up parcellation) is currently required to explore these kinds of questions.

## Discussion

*Connectome* is a multi-purpose toolset which can be used to map, explore, and visualize patterns of ligand–receptor expression in any single-cell dataset. It generalizes the workflow first developed in^1^ and allows adoption by the wider community. The software is open-source on GitHub and includes vignettes to replicate and adapt all analyses discussed. It allows quantification and observation of both fine-grain (single-mechanism, single-celltype-to-celltype vector), and coarse-grain (total signaling family, cross-system) connectivity patterns.

There are a number of clear caveats to the above described techniques. First and foremost, strongly paired ligand and receptor expression is not direct evidence of cell–cell communication. For many ligand–receptor mechanisms, cells must be in direct or very close proximity in order to communicate via that mechanism. Suspension-based single-cell sequencing, however, does not preserve spatial histologic information, and so the techniques described here attempt to make a qualitative portrait of a tissue based on what interactions are *possible*; any robust claim of transduction requires extensive wet-lab experimentation. Although intracellular transduction can be computationally predicted^5^, the complexity of intracellular signaling networks makes this task fundamentally confounded for many ligand–receptor mechanisms.

Secondly, the outputs of *Connectome*, like any ligand–receptor mapping software, are only as good as the ground-truth database against which the original mapping takes place. This is why we have made the ground-truth customizable in this platform. Customization allows removal of extraneous connections with low biologic relevance, or addition of newly-discovered or researcher-hypothesized mechanisms. The software here utilizes the FANTOM5 database without modification, but iterative application has shown that a custom database can be useful for many specific researcher inquiries, in particular in immunology where complex immune cues are of interest.

*Connectome* is designed to be a fundamental tool for single cell researchers, computational biologists, and tissue engineers. It is intended to allow rapid, low-computationally-intensive-access to cell–cell signaling patterns that are present in single-cell data. Our intent is to allow researchers to quickly identify strongly-expressed signaling genes, to find strong pairings between cell types within identified signaling mechanisms, and to condense large amounts of network-level connectivity information into simple, quantitative plots which reflect the structure of tissue systems. We show here that *Connectome* can be applied to individual tissues, paired experimental conditions, and longitudinal datasets. In each case*, Connectome* yields biologically relevant information that can be used to help answer, and inform, specific questions regarding biological systems.

## Supplementary Information

Supplementary Figures.
