# Supplementary material for: Computation and visualization of cell–cell signaling topologies in single-cell systems data using Connectome
Source: Sci Rep. 2022 Mar 9;12:4187. doi: 10.1038/s41598-022-07959-x (PMC8906120; doi:10.1038/s41598-022-07959-x)
Supplement: Supplementary file 1 — Supplementary Figures. [file 41598_2022_7959_MOESM1_ESM.docx]

**Supplement**

**
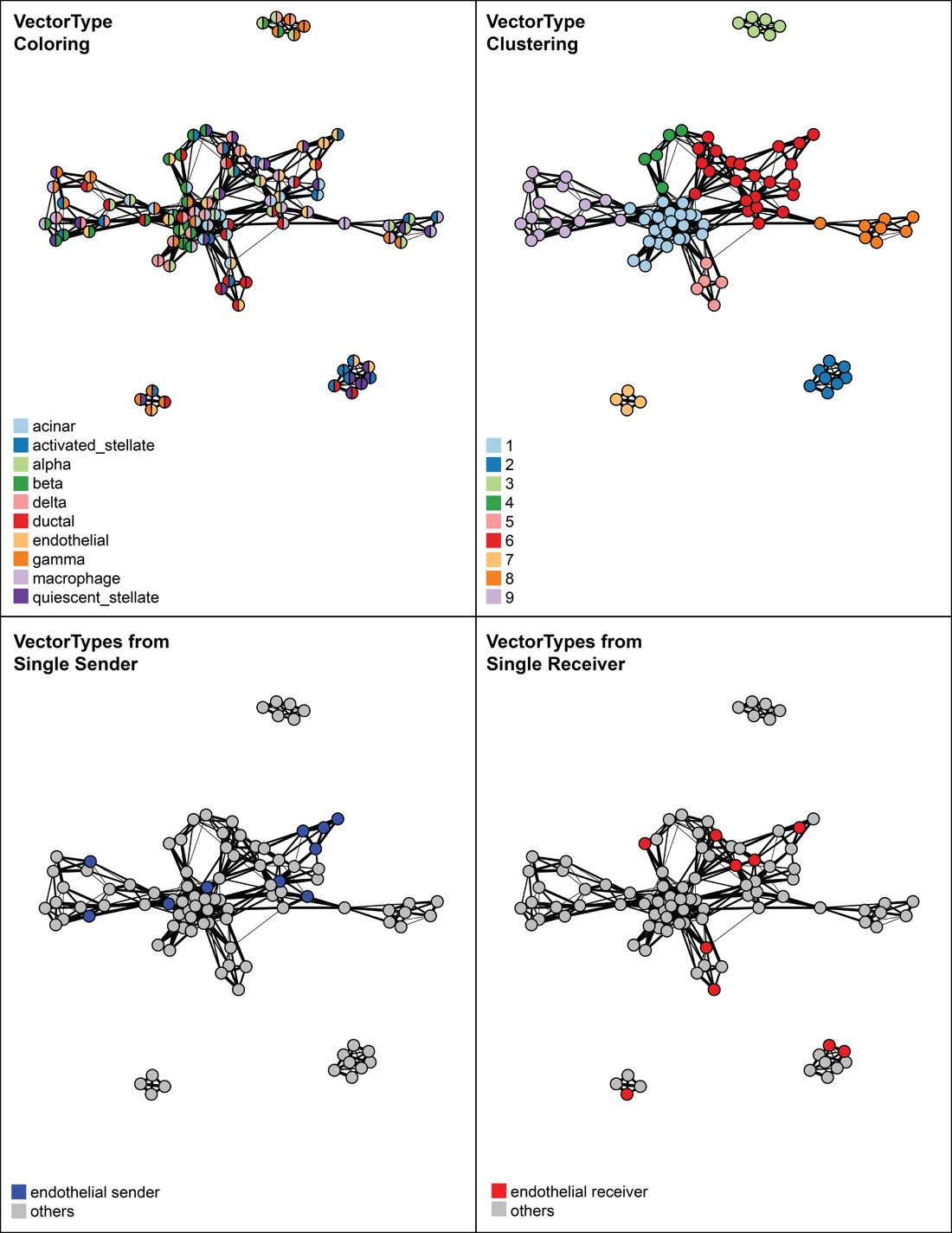
**

**Figure S1: Shared nearest neighbor graph visualization of vector types**. Graphs shown are generated using k=3 in k-nearest neighbor calculation which uses the weight_norm edge attribute of all ligand-receptor mechanisms as features. Each node is a vector type, and an edge is drawn between two nodes that share at least one neighbor. The weights of the edges that reflect the similarity between two nodes are calculated using the rank scheme defined by ^24^. Nodes in the upper left graph are colored in two parts, with the left color representing the sending celltype and the right color representing the receiving. The upper right shows a Louvain clustering of this same graph. As a demonstration, the lower panels emphasize those vectortypes originating only from endothelial cells and landing only on endothelial cells, respectively. Note (i.e. in Cluster 2) that two vectortypes can be highly similar even though they do not all originate from, or land on, the same cell types. This is indicative of substantial overlap in the ligand-receptor mechanism weighting between those two cell types.

**Figure S2: Runtime for connectome computation with and without statistical analysis.** Simulation was run on the panc8 dataset from *SeuratData,* with and without calculation of p-values for each ligand and receptor, on each edge, via a system-wide Wilcoxon Rank Sum test. In both cases, runtime is dependent on both parcellation number and cell number. This simulation was run on a 2.9 GHz 8-core MacBook Pro laptop.
